# Comparison of the Amyloid Load in the Brains of Two Transgenic Alzheimer’s Disease Mouse Models Quantified by Florbetaben Positron Emission Tomography

**DOI:** 10.3389/fnins.2021.699926

**Published:** 2021-10-04

**Authors:** Antje Willuweit, Michael Schöneck, Sarah Schemmert, Philipp Lohmann, Saskia Bremen, Dominik Honold, Nicole Burda, Nan Jiang, Simone Beer, Johannes Ermert, Dieter Willbold, N. Jon Shah, Karl-Josef Langen

**Affiliations:** ^1^Institute of Neuroscience and Medicine (INM-2, INM-4, INM-5, and INM-11), Forschungszentrum Jülich, Jülich, Germany; ^2^Institute of Biological Information Processing, Structural Biochemistry, Forschungszentrum Jülich, Jülich, Germany; ^3^Department of Stereotaxy and Functional Neurosurgery, Faculty of Medicine and University Hospital Cologne, University of Cologne, Cologne, Germany; ^4^Institut für Physikalische Biologie, Heinrich-Heine-Universität Düsseldorf, Düsseldorf, Germany; ^5^JARA-Brain-Translational Medicine, Aachen, Germany; ^6^Department of Neurology, RWTH Aachen University, Aachen, Germany; ^7^Department of Nuclear Medicine, RWTH Aachen University, Aachen, Germany

**Keywords:** Alzheimer’s disease, small animal imaging, florbetaben, amyloid imaging, plaque burden, amyloidosis mouse model, plaque morphology, neuroimaging

## Abstract

Alzheimer’s disease (AD) is characterized by formation of amyloid plaques and neurofibrillary tangles in the brain, which can be mimicked by transgenic mouse models. Here, we report on the characterization of amyloid load in the brains of two transgenic amyloidosis models using positron emission tomography (PET) with florbetaben (FBB), an ^18^F-labeled amyloid PET tracer routinely used in AD patients. Young, middle-aged, and old homozygous APP/PS1 mice (ARTE10), old hemizygous APPswe/PS1ΔE9, and old wild-type control mice were subjected to FBB PET using a small animal PET/computed tomography scanner. After PET, brains were excised, and *ex vivo* autoradiography was performed. Plaque pathology was verified on brain sections with histological methods. Amyloid plaque load increased progressively with age in the cortex and hippocampus of ARTE10 mice, which could be detected with both *in vivo* FBB PET and *ex vivo* autoradiography. FBB retention showed significant differences to wild-type controls already at 9 months of age by both *in vivo* and *ex vivo* analyses. An excellent correlation between data derived from PET and autoradiography could be obtained (*r*_*Pearson*_ = 0.947, *p* < 0.0001). Although amyloid load detected by FBB in the brains of old APPswe/PS1ΔE9 mice was as low as values obtained with young ARTE10 mice, statistically significant discrimination to wild-type animals was reached (*p* < 0.01). In comparison to amyloid burden quantified by histological analysis, FBB retention correlated best with total plaque load and number of congophilic plaques in the brains of both mouse models. In conclusion, the homozygous ARTE10 mouse model showed superior properties over APPswe/PS1ΔE9 mice for FBB small animal amyloid PET imaging. The absolute amount of congophilic dense-cored plaques seems to be the decisive factor for feasibility of amyloidosis models for amyloid PET analysis.

## Introduction

Imaging of amyloid pathology in the brains of Alzheimer’s disease (AD) patients by means of positron emission tomography (PET), and specific radiotracers has been widely used in recent years and plays an important role as inclusion criteria in clinical trials (Villemagne et al., 2017). The first PET tracer developed to specifically bind to amyloid β (Aβ) plaques in AD patients’ brains was named Pittsburgh compound B ([^11^C]PiB). This ^11^C-labeled radiotracer has been shown to possess high affinity and selectivity to fibrillar Aβ in plaques and other Aβ–containing lesions (Cohen et al., 2012). Encouraged by these successful applications, several ^18^F-labeled selective Aβ radiotracers have been developed, such as florbetapir (Amyvid^®^), florbetaben (FBB; NeuraCeq^®^), and flutemetamol (Vizamyl^®^), which successfully replicated the results showing robust differences in tracer retention between patients with AD and age-matched controls (Rowe et al., 2008; Serdons et al., 2009; Wong et al., 2010). All three agents have been approved by the Food and Drug Administration for amyloid imaging and diagnostic use in humans.

Transgenic mice have been established to generate preclinical animal models of AD and to mimic parts of the pathology to study disease mechanisms and new therapeutic strategies. Most mouse models concentrate on only one pathological hallmark of AD, that is, either amyloid or tau pathology. This was achieved by transgenic expression of mutant genes responsible for early-onset familiar AD [e.g., mutated Aβ precursor protein (*APP*) and/or presenilin (*PSEN*)-1 or -2] or frontotemporal dementia (mutated microtubule-associated protein tau), respectively (Ameen-Ali et al., 2017). A few AD models with both amyloid and tau pathology were generated by cross breeding of mouse lines with amyloidosis and tauopathy. In general, transgenic mice harboring mutated *APP*, for instance, with the so-called “Swedish mutation” (APPswe), with or without an additional *PSEN* transgene (e.g., PS1-M146V and PS1ΔE9), do recapitulate the amyloid pathology of AD very well. Accumulation of amyloid in the brains of the mice leads to the progressive deposition of amyloid plaques, dystrophic neurites and accompanying neuroinflammation and the development of cognitive and behavioral deficits upon aging (Ameen-Ali et al., 2017).

Amyloid imaging of transgenic amyloidosis mouse models has been tried with all established amyloid radiotracers, although with varying results (Manook et al., 2012; Poisnel et al., 2012; Rominger et al., 2013; Snellman et al., 2014; Stenzel et al., 2019; Yousefi et al., 2015). The motivation for preclinical PET imaging in AD models is to evaluate newly developed radiotracers and to follow success of new therapeutic options for translation of treatment monitoring into the clinic. Small animal PET imaging using FBB was able to detect Aβ plaques in transgenic amyloidosis models such as APP/PS-21, PS2APP, and NSE-hAPP mice (Brendel et al., 2015; Waldron et al., 2015; Son et al., 2018). However, it has been reported that other well-established amyloidosis models, such as APPswe/PS1ΔE9 mice, expressing comparable APP and PS transgenes and also exhibiting decent and progressive pathology, did not differentiate significantly from wild-type animals and were therefore rated to be unsuitable for FBB PET. It has been reasoned that development of plaques in the reference tissue used for image analysis was one cause for the lacking differentiation from wild-type (Brendel et al., 2015), and additionally, small plaque morphology accounts for low FBB binding (Stenzel et al., 2019). Moreover, studies using the radiotracers [^11^C]PiB and flutemetamol came to the conclusion that APPswe/PS1ΔE9 mice and also the APP model Tg2576 are not suited for amyloid PET imaging, possibly due to their low plaque load (Snellman et al., 2013, 2014). In contrast, the homozygous ARTE10 mouse model expressing both APPswe and PS1-M146V has been described for high binding of [^11^C]PiB and FBB in a proof-of-concept study (Yousefi et al., 2015), and we could show this line to be very suitable for amyloid PET imaging using [^11^C]PiB (Manook et al., 2012). From the literature, it is currently unclear why small animal PET is more successful with one and not the other line. Possible reasons include technical difficulties and differences in amyloid plaque load, plaque morphology, or composition, which could account for lower binding of radiotracers to amyloid fibrils. The latter is highly relevant for clinical amyloid PET, as several reports about confirmed AD patients with negative amyloid PET scan can be found in the literature (Cairns et al., 2009; Rosen et al., 2010; Scholl et al., 2012).

These questions could be answered using preclinical comparative studies with sufficient sample sizes, in which different mouse lines are compared side-by-side, in the same laboratory, under standardized conditions to draw quantitative conclusions about differences in amyloid radiotracer binding. Unfortunately, because of the high efforts and complexity, these kinds of studies are very rare. Notwithstanding, the aims of the present study were (1) to fully characterize the progression of the amyloid deposits in ARTE10 mice by imaging with FBB and (2) to compare this rather new mouse model with the APPswe/PS1ΔE9 line, one of the standard models in preclinical AD research whose feasibility for small animal imaging was questioned. For comparison, we used an optimized imaging protocol for both mouse lines in a reasonably powered and gender-balanced small animal PET imaging study.

## Materials and Methods

### Animals

All animal experiments were performed in accordance with the German Law on the protection of animals (TierSchG §§7–9) and were approved by a local ethics committee [Landesamt für Natur, Umwelt und Verbraucherschutz Nordrhein-Westfalen (LANUV), North Rhine-Westphalia, Germany, Az: 84-02.04.2014.362 and Az: 84-02.04.2011.A359] before start of the study.

Experiments were performed with two different double transgenic mouse lines harboring APP and PS1 transgenes. Homozygous ARTE10 mice [B6.CBA-Tg(Thy1-PSEN1^∗^M146V,-APP^∗^Swe)10Arte, IMSR Cat# TAC:16347, RRID:IMSR_TAC:16347] backcrossed to a congenic C57Bl/6 background and expressing APPswe and PS1-M146V under Thy1.1 regulatory sequences were provided by Taconic Biosciences, Inc. (Germantown, NY, United States). Animals develop an AD-like amyloid pathology beginning at the age of 3 months, which further progresses until 21 months of age (Willuweit et al., 2009).

Hemizygous APPswe/PS1ΔE9 mice (B6.Cg-Tg(APPswe,PSEN1dE9)85Dbo/Mmjax) expressing APPswe and PS1 with deleted exon 9 (PS1ΔE9) were ordered from the Jackson Laboratory (Bar Harbor, ME, United States; MMRRC Cat# 034832-JAX, RRID:MMRRC_034832-JAX) and bred in-house. The line was introduced by Jankowsky et al. (2004) and has been intensively characterized. At 6 months of age, the mice develop Aβ deposits and gliosis, which progressively intensifies with age (Jankowsky et al., 2004). Non-transgenic littermates of this line were used as wild-type controls (WT). All mice were kept in a controlled environment, on a 24-h light–dark cycle (12/12 h) with 54% humidity and a temperature of 22°C. Food and water were available *ad libitum*.

### Study Design

ARTE10 mice at the age of 9, 15, or 21 months and 24-month-old APPswe/PS1ΔE9 or wild-type mice were subjected to FBB PET/computed tomography (CT) measurements. Detailed information about age and gender of each group is given in Table 1. For homozygous ARTE10 mice, a high amyloid load was described to be present already in young animals (Willuweit et al., 2009). Therefore, different ages were included to pick up a possible signal increase. For APPswe/PS1ΔE9, on the other hand, it has been described that FBB retention is very low even at an advanced age (Brendel et al., 2015). Therefore, very old mice were included here, in which the amyloid pathology is already maximally developed in order to achieve a signal at all. The control group was chosen to match the oldest animals of this study. As a stable FBB background signal over age in C57Bl/6 animals has been reported before (Rominger et al., 2013), inclusion of controls of different ages was omitted. Immediately after CT, animals were sacrificed and brains were analyzed by *ex vivo* autoradiography. Afterward, sections were stained for amyloid pathology using Congo red, and plaque load, count, and average size of plaques were quantified.

**TABLE 1:** Overview of mice used for each analysis.

Mouse model	Mean age ± SD (months)	Body weight mean ± SD (g)	Total no. (m + f)	PET	*Ex vivo* autoradiography	Plaque analysis
ARTE10 9 months	9.1 ± 0.4	25.3 ± 4.5	8 (4 + 4)	6 (3 + 3)	6 (3 + 3)	8 (4 + 4)
ARTE10 15 months	15.1 ± 0.1	24.6 ± 4.3	9 (5 + 4)	7 (3 + 4)	6 (2 + 4)	8 (4 + 4)
ARTE10 21 months	21.4 ± 0.6	27.0 ± 3.6	7 (4 + 3)	7 (4 + 3)	7 (4 + 3)	7 (4 + 3)
APPswe/PS1ΔE9	24.1 ± 0.4	26.9 ± 4.7	10 (5 + 5)	9 (4 + 5)	NA	7 (5 + 2)
Wild-type	24.2 ± 0.6	29.8 ± 2.9	9 (5 + 4)	9 (5 + 4)	8 (4 + 4)	NA

*F, female; m, male; NA, not analyzed.*

### Radiosynthesis

Florbetaben was synthesized in an automated synthesis module of Eckert & Ziegler (Berlin, Germany) according to a modified method of Zhang et al. (2005).

[^18^F]fluoride was produced by the ^18^O(p,n)^18^F reaction by bombardment of enriched [^18^O]water with 16.5 MeV protons using a BC1710 cyclotron (The Japan Steel Works Ltd., Shinagawa, Japan) at the INM-5 (Forschungszentrum Jülich, Germany). QMA cartridges (Sep-Pak Accell Plus QMA Carbonate Plus Light, 46 mg sorbent per cartridge) were obtained from Waters (Waters GmbH, Eschborn, Germany) and preconditioned with 1 mL H_2_O directly before use. The labeling mesylate precursor was kindly provided by Piramal Imaging (Berlin, Germany).

[^18^F]fluoride was passed through a QMA cartridge and eluted with K_2_CO_3_ (7 mg) and [2.2.2]Cryptand (22 mg) solved in 600 μL of CH_3_CN/H_2_O (1:1). The solvent was removed at 90°C under a helium stream, and the residue was azeotropically dried with 0.8 mL of anhydrous CH_3_CN twice at 90°C, followed by an additional drying *in vacuo* for 5 min. A solution of the mesylate precursor (8–8.55 mg) in anhydrous CH_3_CN (1.8 mL) was added to the reaction vessel containing the dried [^18^F]fluoride and heated at 120°C for 8 min. After cooling to 60°C, HCl (2 M aqueous solution, 2 mL) was added, and the solution was stirred for another 7.5 min at 110°C. The solution was then cooled to approximately 40°C and transferred to a collector vessel containing 1.2 mL of 2 M NaOH and 0.8 mL of 1 M ammonium formate. The reaction vessel was washed with ethanol (0.5 mL), and the solutions were combined in the collector vessel and a solution. Purification was performed using high-performance liquid chromatography using a Synergi 10 μm Hydro RP 80A column (10 × 250 mm, 10 μm, Phenomenex, Germany), with mobile phase consisting of 60% aqueous ethanol (containing 5 mg/mL sodium ascorbate) at a flow rate 6 mL/min. The fraction with the desired product was collected, diluted, and stirred with water for injection (18 mL) (Braun, Melsungen, Germany). This procedure yields a radiochemical purity exceeding 96% and molar activity of 179–349 GBq/μmol at the end of synthesis.

### Small Animal Positron Emission Tomography/Computed Tomography

Dynamic FBB PET was acquired with ARTE10 animals at the age of 9, 15, or 21 months and APPswe/PS1ΔE9 and WT mice at the age of 24 months. After placement into the INVEON small animal PET/CT scanner (Siemens Preclinical Solutions, Inc.) and a 10 min transmission scan, animals received a slow intravenous bolus injection of 12.2 ± 4 MBq FBB into the lateral tail vein using a tailor-made catheter under isoflurane anesthesia (1.0–1.5%). Dynamic data acquisition was performed in three-dimensional list mode for 120 min starting immediately with injection of the tracer. The emission data were normalized and corrected for scattered and random coincidences, attenuation, decay, and dead time. The resulting sinograms were reconstructed using filtered back-projection into the 35 frames (10 × 5 s, 4 × 10 s, 5 × 30 s, 6 × 60 s, 4 × 300 s, 6 × 600 s), resulting in a reconstructed voxel size of 0.7764 mm × 0.7764 mm × 0.796 mm (matrix size: 128 × 128 × 159). Immediately after the PET scan, animals received a CT scan of 10 min for anatomical coregistration.

After the CT scan, the deeply anesthetized mice were killed by cervical dislocation followed by rapid removal of the brain. Brains were immediately frozen in liquid isopentane (−80°C) and subjected to *ex vivo* autoradiography.

### Positron Emission Tomography Image Analysis

All *in vivo* image data were processed and analyzed with the software PMOD (version 3.7; Pmod Technologies, Zurich, Switzerland). First, manual rigid-body coregistration of individual CT images to a magnetic resonance imaging (MRI) template present in PMOD was performed. Then, manual rigid-body alignment of individual FBB images from early frames (1.3–9.3 min p.i.) to the corresponding individual CT images was accomplished, and resulting coregistration of PET images to the MRI template was visually checked and manually realigned whenever necessary. Volumes of interest (VOIs), as defined in the MRI template, were back-transformed to the original dynamic PET images, and time–activity curves in each VOI were calculated. VOIs were analyzed in averaged frames from late acquisition time points (25–90 min p.i.) and divided by the tracer uptake of the cerebellum or brain stem as reference tissues expressed as standardized uptake value (SUV) ratio.

### Small Animal Magnetic Resonance Imaging

A few animals additionally underwent MRI to make use of the superior soft tissue contrast compared with CT and to carefully check fitting of the coregistration procedure as described above. MRI scans were carried out on a home-integrated 9.4 T small animal scanner (Felder et al., 2017). Using the ^1^H configuration, the static magnetic field was shimmed, and standard calibrations were performed to obtain anatomical and structural images. Mice were anesthetized with 1.5–2.5% isoflurane in oxygen and placed into the animal scanner in which they were measured under continuous isoflurane anesthesia. During the measurement, breathing rate and body temperature were controlled. The acquired MR data were processed using the software PMOD 3.7.

### *Ex vivo* Autoradiography

After PET/CT scan, the freshly frozen right brain hemisphere was cut sagittally into 20 μm sections using a cryostat (Leica CM 3050 S; Leica Microsystems, Wetzlar, Germany), and every 10th slice was exposed to an imaging plate (Fuji Imaging Plate BAS 2025-MS; Fujifilm, Tokyo, Japan) overnight. Freshly prepared sections from FBB mixed with chicken liver paste were also exposed to the imaging plate to yield standards for a tracer calibration curve. The imaging plates were scanned (Fuji BAS Reader 5000; Fujifilm), and resulting autoradiograms were quantitatively evaluated with a pixel size of 25 μm (AIDA version 4.50; Raytest Isotopenmessgeräte GmbH, Straubenhardt, Germany). Circular regions of interest (ROIs), on the basis of a reference staining on neighboring slides, were placed into cortex, cerebellum, and brain stem, and ratio analysis was performed. A larger reference ROI was placed outside the brains to determine the plate background. Integrated densities per region area were measured after background subtraction and used for ROI ratio analyses. At least eight slices were analyzed per animal. Remaining sections were stored frozen for further analyses (see sections below).

### Immunostaining

Immunofluorescence staining against Aβ was performed on 20 μm sagittal frozen brain sections. In brief, room tempered sections were fixed in 4% paraformaldehyde (PFA) in TRIS-buffered saline (TBS), 10 min, room temperature (RT). For antigen retrieval, sections were incubated in 70% formic acid (5 min, RT). Sections were washed three times for 5 min in 1% Triton in TBS (TBST) and incubated with the primary antibody over night at 4°C in a humid chamber (6E10: 1:500, Cat# 803014, RRID:AB_2728527; Bio Legend, San Diego, CA, United States) in TBST with 1% bovine serum albumin (BSA), followed by incubation with Alexa488-labeled secondary anti-mouse antibody (1:300 in TBST+ 1% BSA; Sigma-Aldrich, Darmstadt, Germany). Sections were stained for cell nuclei with DAPI (Sigma-Aldrich) and mounted with Aqua-Poly/Mount (Polysciences Inc., Warrington, PA, United States) after washing in TBS.

### Congo Red Staining

Fluorescence staining with Congo red was performed to stain fibrillar amyloid on frozen brain sections. Frozen 20 μm mouse brain sections were dried in ambient air for 15 min, immersion-fixed in 4% PFA in TBS at RT for 10 min, washed in TBS for 15 min, and rinsed with tap water for 5 min. After incubation in alkaline sodium chloride solution for 20 min, sections were stained in Congo red solution (Amyloid Stain, Congo red-kit, Cat# HT60; Sigma-Aldrich Chemie GmbH, Steinheim, Germany) for 60 min. Then, sections were washed twice in 100% ethanol, 8 min in RotiHistol (Carl Roth GmbH & Co. KG, Karlsruhe, Germany), and mounted with DPX mountant (Sigma-Aldrich).

### Microscopy and Image Analysis

To avoid differences in staining intensity, which might affect measurements, all slides were stained in one batch. Images were acquired with the use of a Zeiss SteREO Lumar V12 (Carl Zeiss AG, Oberkochen, Germany) microscope and the according software (Zeiss AxioVision 6.4 RE) or a Leica LMD6000 microscope (Leica Microsystems GmbH) and the according software (LAS 4.0 software). Quantification of amyloid plaque pathology was performed on overview images of Congo red–stained sagittal brain slices using ImageJ (National Institute of Health, Bethesda, MD, United States) by an experimenter blind to mouse line, genotype, and age group. The % stained area (plaque load), plaque count, and average size of the plaques in the brains of ARTE10 at different ages (in total *n* = 23) and 24-month-old APPswe/PS1ΔE9 (*n* = 7) mice were analyzed in manually drawn ROIs of cortex (5–8 slices/mouse), hippocampus (6 slices/mouse), cerebellum (5–7 slices/mouse), and brain stem (4–6 slices/mouse). Animals for which fewer than four specific ROI-containing sections were available were omitted from the respective analysis.

### Statistical Analysis

All statistical calculations were performed using GraphPad Prism 8 (GraphPad Software, Inc., La Jolla, CA, United States), StatView version 5.0.1 (SAS institute Inc., Cary, NC, United States) or InVivoStat version 3.7.0, [InVivoStat by Simon Bate and Robin Clarke, United Kingdom (Clark et al., 2012)]. All values are represented as mean ± SEM in order to depict the uncertainty of the mean values. The Gaussian distribution of all data was tested using the normality plot in InVivoStat or GraphPad Prism. Normally distributed data were analyzed in the one-way analysis of variance (ANOVA) with Fisher *post hoc* analysis. Correlation analysis was performed using the Pearson parametric correlation test and expressed as pairwise Pearson correlation coefficient (*r*). *p* < 0.05 was considered to indicate significant statistical differences in all tests.

## Results

### Positron Emission Tomography Imaging With Florbetaben in ARTE10 Mice

**FIGURE 1:**
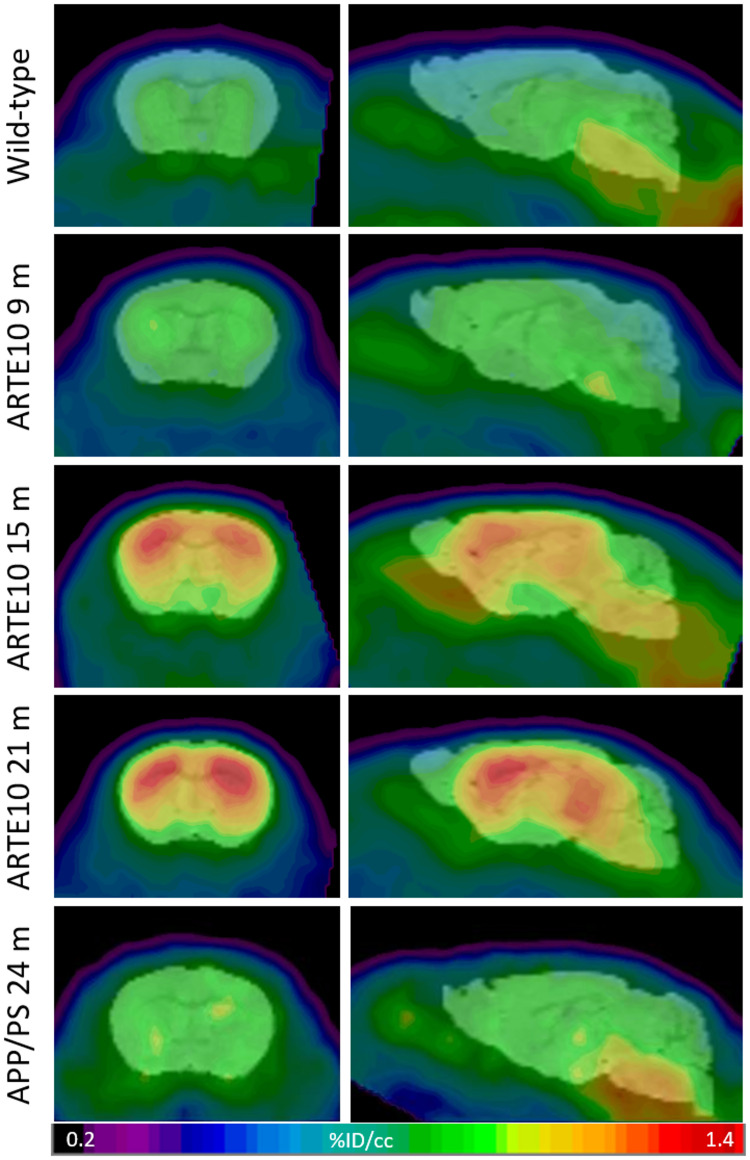
Progressive FBB retention in the brains of ARTE10 and APP/PS mice. Wild-type control animals, homozygous ARTE10 mice at the age of 9, 15, or 21 months, and 24-month-old APPswe/PS1ΔE9 (APP/PS) were subjected to FBB PET. Coronal (left) and sagittal (right) views of cranial tracer retention (% injected dose per volume, %ID/mL) averaged over 25–90 min post-injection. Representative animals are shown coregistered to a mouse brain MRI atlas.

ARTE10 mice of different ages were subjected to FBB PET in order to validate the ARTE10 mouse model concerning its suitability for amyloid PET imaging. With increasing age, more FBB retention was observed in the brains of homozygous ARTE10, whereas in 24-month-old WT mice, only a background signal could be observed (Figure 1 and Supplementary Figure 1). Progressive FBB signal was mainly observed in the cortex and hippocampus, followed by thalamus at later stages, whereas the cerebellum remains nearly free of FBB. White matter binding of FBB, as observed already by others (Rominger et al., 2013), could be observed in all animals including WT.

**FIGURE 2:**
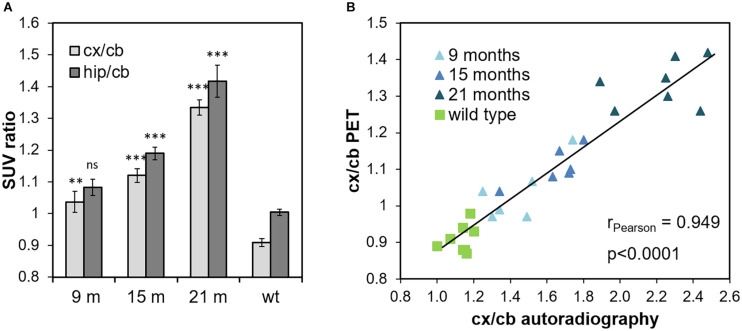
Quantification of FBB retention measured by PET and *ex vivo* autoradiography. **(A)** *In vivo* tracer retention was quantified in cortical and hippocampal VOIs with cerebellum as reference region and expressed as cortex-to-cerebellum (cx/cb) and hippocampus-to-cerebellum ratios (hip/cb), respectively. Data are expressed as mean ± SEM; ns, not significant, **p < 0.001, ***p < 0.0001 vs. wild-type (wt). **(B)** Volumetric quantification of cortex-to-cerebellum ratios (cx/cb) as measured by PET and autoradiography in the very same animal, respectively, are plotted against each other (linear regression line, *R*^2^ = 0.9). Each symbol represents one ARTE10 mouse of the respective age groups (triangle) or a wild-type mouse (square).

Quantification of the FBB PET signals in cortical and hippocampal VOIs was done using the cerebellum as reference tissue as suggested before (Manook et al., 2012). SUV ratios (SUVR_cb_) in both tissues of ARTE10 mice showed a progressive increase with increasing age (Figure 2A). Already in 9-month-old ARTE10 mice, and at all ages thereafter, the SUVR_cb_ of FBB retention in the cortex was significantly different to WT animals (one-way ANOVA *F*_3_,_25_ = 69.3, *p* > 0.0001, Fisher *post hoc* analysis vs. WT: 9 months *p* = 0.0004, 15 months *p* > 0.0001, 21 months *p* > 0.0001). In the hippocampus, FBB SUVR_cb_ reached statistical significance compared with WT starting at 15 months of age [one-way ANOVA *F*_3_,_25_ = 40.4, *p* > 0.0001, Fisher *post hoc* analysis vs. WT: 9 months *p* = 0.064 (not statistically significant), 15 months *p* > 0.0001, 21 months *p* > 0.0001]. Progression of FBB retention in the cortex started with 114 ± 4% relative to WT with 9 months and rose up to 147 ± 3% in 21-month-old ARTE10 mice (Fisher *post hoc* analysis: 9 vs. 15 months *p* = 0.0187, 9 vs. 21 months *p* < 0.0001, 15 vs. 21 months *p* < 0.0001). FBB retention in the hippocampus progressed from 108 ± 3% (at 9 months) to 141 ± 5% (at 21 months) relative to WT levels (Fisher *post hoc* analysis: 9 vs. 15 months *p* = 0.02, 9 vs. 21 months *p* < 0.0001, 15 vs. 21 months *p* < 0.0001).

### Correlation Between Florbetaben Positron Emission Tomography and *ex vivo* Autoradiography

**FIGURE 3:**
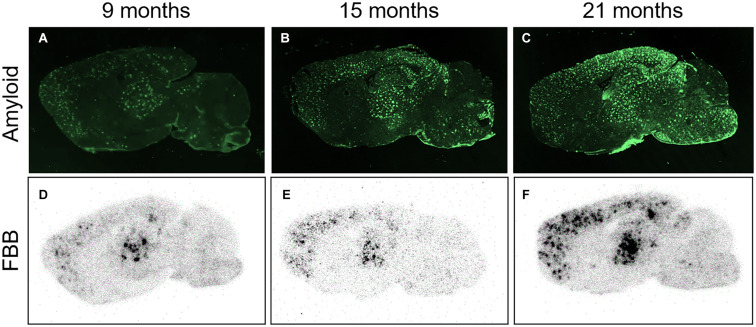
FBB *ex vivo* autoradiography in comparison to amyloid plaque load. **(A–C)** Photographs of parasagittal brain sections from transgenic ARTE10 mice stained by immunofluorescence against Aβ plaques (antibody 6E10) at 9 **(A)**, 15 **(B)**, and 21 months of age **(C)**. Plaque load is increasing with age in ARTE10 mouse brain. **(D–F)** Increasing plaque load is also picked up by FBB *ex vivo* autoradiography on adjacent brain sections of 9- **(D)**, 15- **(F)**, and 21-month-old ARTE10 mice **(F)**.

Right after the PET measurements, the animals were sacrificed, one brain hemisphere was cut, and every 10th section was subjected to *ex vivo* autoradiography. Autoradiograms of ARTE10 brain sections displayed radiotracer binding in the brain with a characteristic plaque-like pattern (Figure 3). This pattern was mainly visible in the cortex, hippocampus, and thalamus of transgenic mice and increased with age of the animals. Comparison to immunofluorescence staining against Aβ on adjacent sections confirmed these brain regions to contain progressive plaque pathology. The cerebellum of ARTE10 mice stayed virtually free of amyloid pathology and displayed little FBB binding.

Volumetric quantification of FBB binding in ROIs on the autoradiograms verified the progressive increase of FBB signals in the cortex, hippocampus, and thalamus (Figure 2B and data not shown). Using again the cerebellum as reference region, cortex-to-cerebellum ratios were calculated and compared with those obtained by PET analyses of the very same animals, in order to validate the latter by the autoradiographic method with higher resolution (Figure 2B). Correlation analysis yielded an excellent correlation between FBB binding quantified by *in vivo* PET and *ex vivo* autoradiography with a Pearson correlation coefficient of 0.949 (*p* < 0.0001).

### Comparison of Florbetaben Positron Emission Tomography in ARTE10 and APPswe/PS1ΔE9 Mice

Florbetaben PET analyses with old APPswe/PS1ΔE9 mice were performed using the same volumetric reference tissue approach as described above for the ARTE10 model. As the cerebellum has been described to develop amyloid pathology in old APPswe/PS1ΔE9 mice (Brendel et al., 2015), a different reference tissue was required. Based on Aβ immunostainings, the brain stem was chosen as the suitable reference tissue for this mouse line as it contained nearly no amyloid, even in very old animals (data not shown). For a comparison of this mouse line to ARTE10 mice, the brain stem seemed to be a good compromise, although some amyloid pathology in brain stem could be observed in old ARTE10 mice (Figure 3).

**FIGURE 4:**
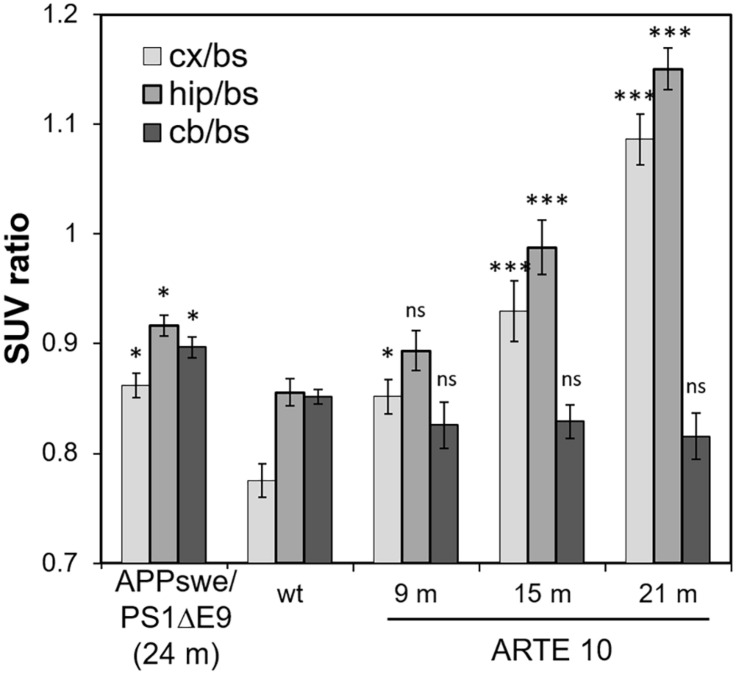
Comparison of FBB retention in the brains of ARTE10 and APPswe/PS1ΔE9 mice. For comparison of both mouse lines, FBB retention was quantified in the cortex (cx), hippocampus (hip), and cerebellum (cb) with brain stem (bs) as reference region and expressed as SUVR. Data are expressed as mean ± SEM; ns, not significant, *p < 0.05, ***p < 0.0001 vs. wild-type (wt).

Reanalysis of FBB retention in the cortex and hippocampus of ARTE10 mice with brain stem as reference tissue resulted in the same progressive FBB signal with increasing age as demonstrated in Figure 4. FBB retention progressed in the cortex of ARTE10 mice from 110 ± 2% relative to WT at 9 months up to 140 ± 3% at 21 months of age. In the hippocampus, progression started at 104 ± 2% (9 months) and reached 134 ± 2% of WT levels (21 months).

Florbetaben retention in the cortex and hippocampus of 24-month-old APPswe/PS1ΔE9 mice reached 111 ± 1% and 107 ± 1% relative to WT, respectively, which was in the range of FBB binding in 9-month-old ARTE10 mice. SUVRs to brain stem as reference (SUVR_bs_) of APPswe/PS1ΔE9 in both cortex and hippocampus were significantly higher than those of WT animals (cortex: one-way ANOVA *F*_4_,_33_ = 39.88, *p* > 0.0001, Fisher *post hoc* analysis vs. WT: APPswe/PS1ΔE9 *p* = 0.0011, 9 months *p* = 0.009, 15 months *p* > 0.0001, 21 months *p* > 0.0001; hippocampus: one-way ANOVA *F*_4_,_33_ = 39.88, *p* > 0.0001, Fisher *post hoc* analysis vs. WT: APPswe/PS1ΔE9 *p* = 0.0085, 9 months statistically not significant *p* = 0.0971, 15 months *p* > 0.0001, 21 months *p* > 0.0001). Analysis of SUVR_bs_ of cerebellar VOIs resulted in a significant difference to WT in APPswe/PS1ΔE9 cerebellum but not in those of ARTE10 mice at any age [Figure 4; one-way ANOVA *F*_4_,_33_ = 5.68, *p* = 0.0014, Fisher *post hoc* analysis vs. WT: APPswe/PS1ΔE9 *p* = 0.0165 (not statistically significant), 9 months *p* = 0.215 (not statistically significant), 15 months *p* = 0.262 (not statistically significant), 21 months *p* = 0.095 (not statistically significant)].

### Amyloid Plaque Pathology in ARTE10 and APPswe/PS1ΔE9 Mice

**FIGURE 5:**
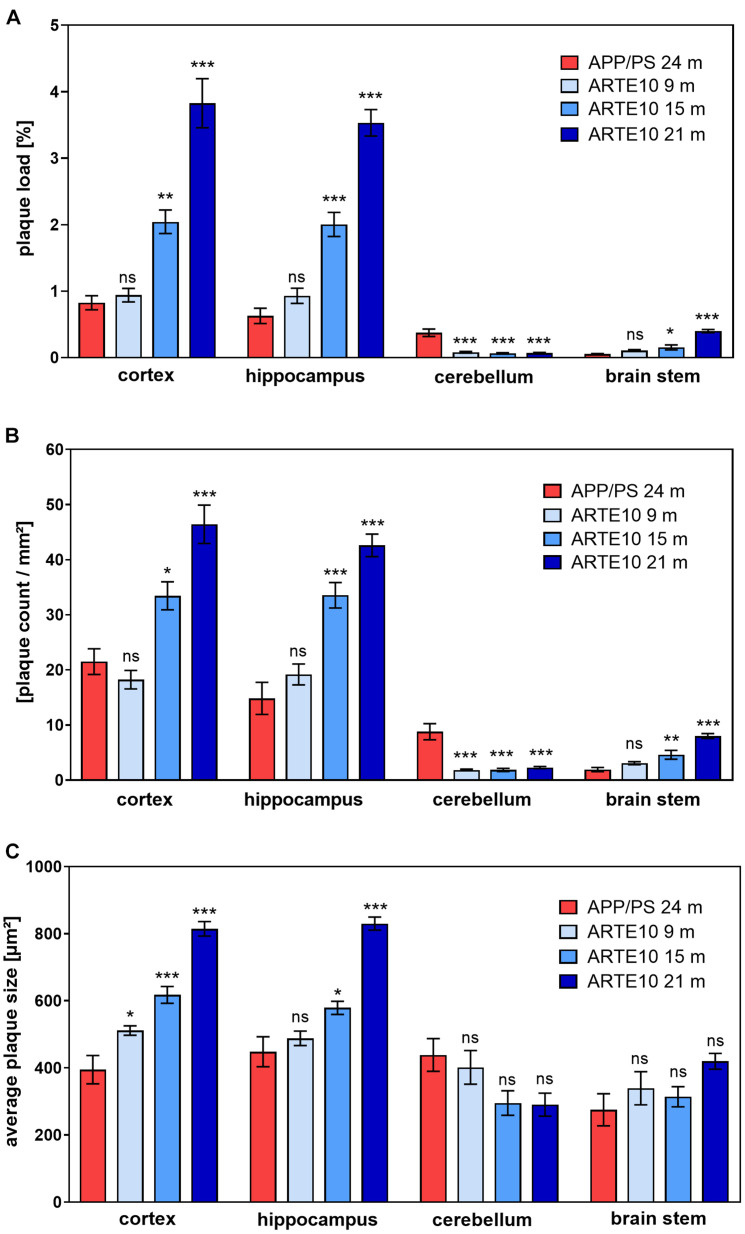
Quantification of amyloid plaque pathology in the brains of ARTE10 and APPswe/PS1ΔE9 mice. Plaque load **(A)**, number **(B)**, and average size **(C)** of amyloid plaques were quantified on Congo red–stained sagittal brain slices from 24-month-old APPswe/PS1ΔE9 (APP/PS) and ARTE10 mice of different ages (9–21 months). Data are expressed as mean ± SEM; ns, not significant, *p < 0.05, **p < 0.001, ***p < 0.0001 vs. APP/PS.

To verify the different amyloid load in the mouse models measured by *in vivo* PET, a histological plaque analysis was conducted on brain slices of ARTE10 and APPswe/PS1ΔE9 mice. Amyloid pathology was stained by Congo red, a dye known for its binding to amyloid fibrils, and % plaque load, the number of plaques per area, and the average size of plaques were quantified (Figure 5). In the cortex and hippocampus of 24-month-old APPswe/PS1ΔE9 mice, the plaque load and plaque count were found to be in the same range of those of 9-month-old ARTE10 mice (Figures 5A,B). Also, average size of the plaques differed only in the cortex, where 9-month-old ARTE10 mice showed slightly bigger plaques than the APPswe/PS1ΔE9 mice (Figure 5C). At the age of 15 and 21 months, plaque load, count, and size increased progressively in the cortex and hippocampus of ARTE10 mice and were statistically significant to APPswe/PS1ΔE9 mice at all times (one-way ANOVA cortex: plaque load, *F*_3_,_26_ = 42.35, *p* > 0.0001; plaque count, *F*_3_,_26_ = 24.77, *p* > 0.0001; plaque size, *F*_3_,_26_ = 41.91, *p* > 0.0001; hippocampus: plaque load, *F*_3_,_26_ = 67.01, *p* > 0.0001; plaque count, *F*_3_,_26_ = 29.93, *p* > 0.0001; plaque size, *F*_3_,_26_ = 36.71, *p* > 0.0001; for *post hoc* analysis, see Table 2). As expected, APPswe/PS1ΔE9 mice displayed amyloid plaques in the cerebellum. Quantification resulted in plaque load and plaque count values approximately 50% of cortex values (Figure 5). In contrast, ARTE10 mice had neglectable amyloid plaque pathology in the cerebellum, which was significantly different to that of APPswe/PS1ΔE9 mice (one-way ANOVA cerebellum: plaque load, *F*_3_,_25_ = 26.88, *p* > 0.0001; plaque count, *F*_3_,_25_ = 21.15, *p* > 0.0001; for *post hoc* analysis, see Table 2). While plaque load in the cerebellum of ARTE10 mice was unchanged over time, plaque count showed a small but statistically non-significant increase with age. However, in the brain stem, APPswe/PS1ΔE9 mice had nearly no detectable plaques, but ARTE10 mice developed amyloid plaque pathology over time, which reached statistical significance to APPswe/PS1ΔE9 mice at 15 and 21 months of age (one-way ANOVA brain stem: plaque load, *F*_3_,_22_ = 45.50, *p* > 0.0001; plaque count, *F*_3_,_22_ = 28.35, *p* > 0.0001; for *post hoc* analysis, see Table 2). No differences in plaque size between the groups were found in the cerebellum or brain stem (one-way ANOVA cerebellum: *F*_3_,_25_ = 2.879, *p* = 0.056; brain stem, *F*_3_,_22_ = 2.155, *p* = 0.1222).

**TABLE 2:** Statistical analyses of plaque quantification on Congo red–stained brain slices from APPswe/PS1ΔE9 (APP/PS, 24 months) and ARTE10 mice of different ages (9–21 months).

	Plaque	Plaque	Plaque
	load	count	size
**Cortex**			
APP/PS vs. ARTE10 9 months	0.7024	0.3713	0.0049^*^
APP/PS vs. ARTE10 15 months	0.0003^**^	0.0027^*^	< 0.0001^***^
APP/PS vs. ARTE10 21 months	< 0.0001^***^	< 0.0001^***^	< 0.0001^***^
ARTE10 9 months vs. ARTE10 15 months	0.0007^**^	0.0002^**^	0.0074^*^
ARTE10 9 months vs. ARTE10 21 months	< 0.0001^***^	< 0.0001^***^	< 0.0001^***^
ARTE10 15 months vs. ARTE10 21 months	< 0.0001^***^	0.0013^*^	< 0.0001^***^
**Hippocampus**			
APP/PS vs. ARTE10 9 months	0.1876	0.1943	0.3144
APP/PS vs. ARTE10 15 months	< 0.0001^***^	< 0.0001^***^	0.0025^*^
APP/PS vs. ARTE10 21 months	< 0.0001^***^	< 0.0001^***^	< 0.0001^***^
ARTE10 9 months vs. ARTE10 15 months	< 0.0001^***^	0.0001^**^	0.0235^*^
ARTE10 9 months vs. ARTE10 21 months	< 0.0001^***^	< 0.0001^***^	< 0.0001^***^
ARTE10 15 months vs. ARTE10 21 months	< 0.0001^***^	0.01^*^	< 0.0001^***^
**Cerebellum**			
APP/PS vs. ARTE10 9 months	< 0.0001^***^	< 0.0001^***^	NA
APP/PS vs. ARTE10 15 months	< 0.0001^***^	< 0.0001^***^	NA
APP/PS vs. ARTE10 21 months	< 0.0001^***^	< 0.0001^***^	NA
ARTE10 9 months vs. ARTE10 15 months	0.6609	0.9848	NA
ARTE10 9 months vs. ARTE10 21 months	0.7705	0.6641	NA
ARTE10 15 months vs. ARTE10 21 months	0.8864	0.6876	NA
**Brain stem**			
APP/PS vs. ARTE10 9 months	0.09	0.1008	NA
APP/PS vs. ARTE10 15 months	0.0041^*^	0.0009^**^	NA
APP/PS vs. ARTE10 21 months	< 0.0001^***^	< 0.0001^***^	NA
ARTE10 9 months vs. ARTE10 15 months	0.1469	0.0385^*^	NA
ARTE10 9 months vs. ARTE10 21 months	< 0.0001^***^	< 0.0001^***^	NA
ARTE10 15 months vs. ARTE10 21 months	< 0.0001^***^	0.0001^**^	NA

*Shown are results of ANOVA *post hoc* analysis by Fisher multiple comparisons.*

^*^p < 0.05, ^**^p < 0.001, ^***^p < 0.0001.

*NA, not analyzed.*

### Correlation Between Florbetaben Positron Emission Tomography and Histological Plaque Analysis

**FIGURE 6:**
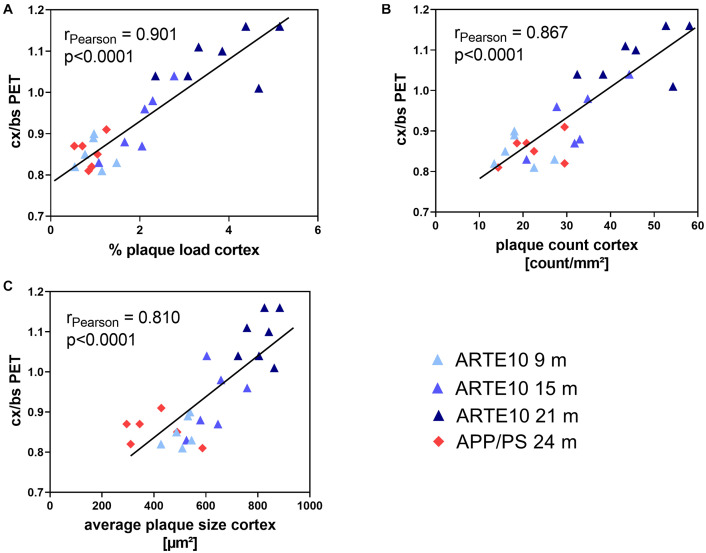
Correlation between *ex vivo* histological amyloid plaque pathology and *in vivo* amyloid load as determined by PET analysis. Plaque load **(A)**, number **(B)**, and average size **(C)** of amyloid plaques in the cortex as quantified using Congo red–stained brain slices from 24-month-old APPswe/PS1ΔE9 (APP/PS) and ARTE10 mice of different ages (9–21 months) are correlated with SUVR of FBB retention in the cortex referenced to the brain stem (cx/bs). Each data point represents the value of a single animal. Pearson correlation coefficient *r* and *p*-values of the statistical analysis are given for each data set.

A pairwise correlation analysis was performed to compare the values of amyloid PET measurements to actual amyloid plaque pathology as quantified from Congo red–stained histological sections. For the cortex and hippocampus, very high and significant correlations were found between FBB binding quantified by *in vivo* PET and histological plaque load, plaque count, and average plaque size of each animal. Pearson correlation coefficients in these brain regions ranged between 0.81 and 0.94 (Figure 6 and Table 3). In the cerebellum, FBB retention correlated weakly with histological plaque load and count, but not with average plaque size in this brain region (Table 3).

**TABLE 3:** Correlation between SUVRs referenced to brainstem from PET analyses and % plaque load, plaque count, and average plaque size as analyzed with Congo red–stained brain slices of the same animals.

	Plaque load	Plaque count	Plaque size
SUVR cx/bs	0.9012^***^	0.8666^***^	0.8100^***^
SUVR hip/bs	0.9424^***^	0.8637^***^	0.8257^***^
SUVR cb/bs	0.5853^*^	0.5982^*^	0.2266

*Pearson correlation coefficient (*r*) is shown for each pairwise correlation.*

*Bs, brain stem; cb, cerebellum; cx, cortex; hip, hippocampus.*

^*^p < 0.05, ^***^p < 0.0001.

## Discussion

Mouse models of AD are a widely used preclinical tool to study disease mechanisms and new therapeutic options. Small animal PET using amyloid radiotracers aims at monitoring amyloid pathology during preclinical therapeutic studies. In addition, suitable animal models are needed to develop and validate new radiotracers. In any case, a proper characterization of the amyloid pathology and its progression in relation to the imaging characteristics of a given model are crucial for meaningful PET studies. It has been reported using established radiotracers such as FBB, [^11^C]PiB, and flutemetamol that some mouse models of amyloidosis are more suitable for imaging of amyloid load than others (Snellman et al., 2013, 2014; Brendel et al., 2015). To date, the exact reasons for these different results are still unclear. It has been speculated that differences in plaque size or total plaque load might be explanations for low retention of amyloid tracers in the brain. Others accounted technical reasons, for example, development of plaque pathology in the reference region for insufficient differentiation to wild-type mice (Brendel et al., 2015). However, comparison of the amount of radiotracer binding in different mouse models and conclusions about their suitability can only be drawn from studies in which the lines have been analyzed in the same laboratory, under standardized conditions and with optimized protocols for all lines. Otherwise, differences in protocols and image analysis pipelines are confounding factors for absolute comparability. In addition, use of sufficient, gender-balanced numbers of animals is of importance, as interindividual variations in plaque pathology, including sex differences, are quite high in some lines (Jankowsky and Zheng, 2017) and may lead to inaccurate conclusions.

For these reasons, we have carried out a small animal PET imaging study to thoroughly characterize the homozygous ARTE10 mouse model for its amyloid imaging properties using the amyloid PET tracer FBB and compare it with the widely used APPswe/PS1ΔE9 mouse model. Special emphasis was put on sufficient numbers and a sex-balanced design of the study. Homozygous ARTE10 mice have been reported to develop robust amyloid plaque pathology in the brain, starting at the age of 3 months and progressing further until 20 months of age with 100% penetrance of the phenotype (Willuweit et al., 2009). Here, we were able to successfully image the progressive amyloid pathology in the brain of this mouse model over increasing age for the first time by the use of FBB PET. A volumetric ratio analysis was chosen to quantify FBB retention in cerebral VOIs with cerebellum as reference tissue, as this brain region is virtually free of plaque pathology in this model. This has been reported by us before (Manook et al., 2012) and could be confirmed here by immunostaining (Figure 3) and plaque analysis (Figure 5). The used SUVR PET analysis approach was validated by *ex vivo* autoradiography on brain sections from the very same animals, which showed an excellent correlation to values yielded from *in vivo* FBB PET. Also, *ex vivo* autoradiograms confirmed retention of FBB in brain areas with high plaque load.

As small animal PET is prone to technical and experimental errors, validation of the imaging protocols is very crucial. The challenges for amyloid imaging in small animals are mainly a low resolution of the PET scanners in relation to the brain size and unspecific binding of the tracers outside the brain. These lead to spillover and partial volume effects especially in small areas of interest and limit the size of the brain regions to be analyzed. To address this issue, exact coregistration procedures to MRI or CT images are crucial to correctly define the brain area of interest. As slight deviations lead to overestimations or underestimations in single brain areas, quality control of the coregistration procedures is very important. In this study, correct coregistration was validated using CT and MRI measurements of a subset of animals, and *ex vivo* autoradiographic analyses were performed, which are able to pick up radiotracer signals with much higher resolution. Thus, the excellent correlation between *ex vivo* autoradiography and *in vivo* PET from the very same animals validated the precision of our PET imaging protocol. Nevertheless, the PET imaging signal was underestimated in comparison to autoradiography as the gold standard (Figure 2B). This is most likely due to the partial volume effect, which has been described by others before and can be minimized using a partial volume correction procedure (Brendel et al., 2014). Also, only sections from one brain hemisphere were used for autoradiographic analyses, whereas PET analyses were done on the whole brain. Asymmetry of amyloid load between both brain hemispheres has been described for another mouse model (Rominger et al., 2013). Given the high correlation between *ex vivo* autoradiography and *in vivo* PET in this study, this phenomenon seems to be very unlikely but cannot be excluded completely.

Analysis of amyloid load in ARTE10 mice by FBB PET revealed a differentiation to WT mice in the cortex already at the age of 9 months. The amyloid load in the cortex increased steadily and reached 147% in relation to wild-type values at the age of 21 months. In comparison to what has been reported for FBB PET with other mouse models of amyloidosis, these values are as high as those described for models with high FBB retention (homozygous PS2APP and APP-PS21 mice) and clearly superior to the single transgenic lines expressing only APPswe or Aβ42 (Rominger et al., 2013; Brendel et al., 2015; Waldron et al., 2015; Son et al., 2018). In the hippocampus, differentiation between ARTE10 and WT was reached later, with 15 months of age, which might be attributed mainly to partial volume effects of the PET analysis as the hippocampal VOIs are relatively small. In addition, slightly smaller plaque sizes in the hippocampus as compared with the cortex in 9-month-old ARTE10 mice (Figure 5C) could possibly contribute also to lower FBB retention in this brain region.

Florbetaben retention in the cortex and hippocampus of ARTE10 mice progressed significantly between 9 and 21 months of age, following the statistically significant increase of plaque load, number of plaques, and average plaque size as determined by histological staining. For preclinical studies in which FBB PET could be used to monitor treatment success of an antiamyloid therapy, a progressive radiotracer retention with increasing age is advantageous, as it determines the time window for detection of treatment effects. Thus, given the early onset, robust signal, and progressive tracer retention, the homozygous ARTE10 mouse model in combination with FBB PET represents an ideal combination for future preclinical treatment studies.

Our results of FBB retention in 21-month-old homozygous ARTE10 mice are in very good agreement with those generated by Yousefi et al. (2015), who compared retention of FIBT, FBB and [^11^C]PiB in a small cohort of 24-month-old homozygous ARTE10 mice. The FIBT binding patterns were found comparable to those of FBB and [^11^C]PiB (Yousefi et al., 2011a, 2015), and a comparable binding pattern was also demonstrated between IBT and [^11^C]PiB (Yousefi et al., 2011b). Binding characteristics to the brains of hemizygous and homozygous ARTE10 mice were best characterized for [^11^C]PiB PET in a thorough feasibility study in which imaging capabilities of young and old homozygous ARTE10 were compared with old hemizygous mice (Manook et al., 2012). Even voxel-wise analyses were feasible using hemizygous ARTE10 mice (von Reutern et al., 2013). However, a direct comparison of the ARTE10 line to other, more established AD mouse models had not been carried out before.

Therefore, the second aim of the current study was to compare the ARTE10 model to the APPswe/PS1ΔE9 mice. The APPswe/PS1ΔE9 mouse line was originally described by Jankowsky et al. (2004) and has been characterized very extensively. Like ARTE10 mice, the model carries two transgenes expressing APPswe and PS1ΔE9, which leads to higher production of Aβ42 and a progressive plaque pathology in the brain starting at approximately 6 months of age (Jankowsky et al., 2004). This line is one of the standard mouse models of amyloidosis and has been frequently used to study AD pathophysiology and for proof-of-concept studies of new therapeutic options (Frost et al., 2015; Yanagisawa et al., 2015; van Groen et al., 2017). We have used our FBB PET imaging protocol, which was established in ARTE10, to analyze the amyloid load in old APPswe/PS1ΔE9 mice. As it was described that APPswe/PS1ΔE9 do develop plaque pathology in the cerebellum (Brendel et al., 2015; Stenzel et al., 2019), we used the brain stem as reference region for PET analysis instead. By histological plaque quantification, we were able to confirm a decent plaque burden in the cerebellum and no plaque pathology in the brain stem in this model (Figure 5). PET analysis revealed FBB retention in the cortex, hippocampus, and cerebellum of APPswe/PS1ΔE9 mice, which was statistically significant from WT in all three brain regions. This observation is in contrast to that of Brendel et al. (2015), who could not differentiate FBB retention in the cortex of 24-month-old APPswe/PS1ΔE9 from that of wild-type mice. This discrepancy is most likely explained by their use of cerebellum as reference region. In another study, FBB PET was done with 12-month-old APPswe/PS1ΔE9 and yielded significantly higher FBB signals in the hippocampus of transgenic vs. wild-type mice (Stenzel et al., 2019) even despite using cerebellum as reference region.

In order to compare FBB retention in the cortex, hippocampus, and cerebellum of both mouse models, ARTE10 and APPswe/PS1ΔE9, PET data from ARTE10 mice were reanalyzed based on the brain stem as reference tissue. Therefore, the obtained SUVR_bs_ values were lower than previously calculated SUVR_cb_ values, which could be related to high unspecific binding of FBB in this white matter containing brain region (Rominger et al., 2013). Irrespective of this, however, the relative differences of FBB retention found in the cortex and hippocampus of ARTE10 mice of increasing age and in comparison to WT were similar to the previous analysis. FBB retention in the cerebellum of ARTE10 mice was found to be low and in the range of WT levels matching the actually low plaque burden in this brain region. There was even a trend for decreasing SUVR_bs_ values in the cerebellum of ARTE10 mice with increasing age. This could be attributed to the development of plaque pathology in the brain stem upon aging, which we could actually confirm by histological quantification. However, plaque burden in the brain stem of ARTE10 mice was rather low in comparison to that of other brain regions, except cerebellum, why the use of brain stem as reference region in this model is still justifiable. Nevertheless, this example shows how important the choice of the right reference region is in this ratio-based PET analysis and that a compromise has to be found when comparing different models.

Florbetaben retention in the cortex and hippocampus of 24-month-old APPswe/PS1ΔE9 was found to be in the range of 9-month-old ARTE10 mice. As ARTE10 mice are homozygous for the transgene in contrast to APPswe/PS1ΔE9 mice, a generally higher plaque pathology could be causative for this observation. In order to answer the question whether the signal obtained by *in vivo* FBB PET is indeed based on a lower plaque burden of old APPswe/PS1ΔE9 in comparison to old ARTE10 mice, we performed histological in-depth plaque quantification analyses of the brain tissue. For detection of amyloid plaques, we chose staining with Congo red as this classical amyloid stain is known to be specific for fibrillary Aβ present in dense plaques and expected to mirror binding of amyloid tracers better than immunostaining, which stains also non-fibrillar Aβ. Actually, plaque quantification revealed both % load and number of dense plaques in the cortex and hippocampus of APPswe/PS1ΔE9 mice to be in the range of 9-month-old ARTE10 mice. ARTE10 mice displayed generally bigger plaques than APPswe/PS1ΔE9 mice, which was especially evident with increasing age and true for the cortex and hippocampus but not cerebellum and brain stem. Therefore, it seems that the numbers and total plaque load account most for high FBB retention in the brains of ARTE10 mice. This is also reflected by correlation analyses, which showed the highest correlation between % plaque load or plaque count and FBB retention in the cortex or hippocampus of all mice (Figure 6 and Table 3).

APPswe/PS1ΔE9 mice have been described to deposit high plaque load of diffuse amyloid, which can be stained by antibodies against Aβ, but substantially lower amount of dense-cored plaques that contain fibrillary Aβ and are positive for dyes such as thioflavin S (Frost et al., 2012). In contrast, ARTE10 mice develop mainly dense-cored plaques, resulting in similar plaque load values after staining with either Aβ antibodies or thioflavin S (Willuweit et al., 2009; Manook et al., 2012). It is known that diffuse plaques are not congophilic as they contain loosely distributed Aβ bundles (Yamaguchi et al., 1988, 1991). Non-cored plaques have also been called cotton wool plaques and were found in the brains of AD patients harboring PS mutations, notably the PS1ΔE9 mutation among others, but not the PS1-M146V mutation, which is present in ARTE10 mice (Larner and Doran, 2006). Cotton wool plaques are a very rare form of plaques but the predominant type in individuals with PS1ΔE9 mutation and early-onset AD. However, there were also reports about sporadic late-onset AD patients harboring numerous neocortical cotton wool plaques admixed with diffuse plaques and plaques with dense amyloid core (Le et al., 2001; Yokota et al., 2003). Generally, diffuse and dense-cored plaques can be found in the brains of both early- and sporadic late-onset AD, as the type of lesion largely depends on the involved brain area and stage of the disease (Duyckaerts et al., 2009). The common view is that diffuse plaques represent an early stage of Aβ deposition, which can mature further into classic and compact cored plaques. Accordingly, diffuse plaques are often present in cases without cognitive impairments.

Containing mainly diffuse and non-cored Aβ deposits, it is conceivable that a lower amount of congophilic, dense-cored plaques is the reason for low FBB binding in the brains of APPswe/PS1ΔE9 mice in comparison to ARTE10 mice. A similar hypothesis was put forward by Snellman et al. (2014) using flutemetamol in APPswe/PS1ΔE9 mice. For [^11^C]PiB, however, binding to diffuse plaques has been demonstrated using the fluorescent derivative 6-CN-PiB, which has identical binding properties as [^11^C]PiB (Mathis et al., 2017). In any case, systematic proof for lower binding of FBB to non-dense plaques is missing so far. This phenomenon is of clinical relevance as AD patients harboring PS mutations and/or cotton wool plaques are expected to appear amyloid negative in the amyloid PET scan. Negative PET scans have also been described for [^11^C]PiB in individuals with confirmed diagnosis of AD (Cairns et al., 2009; Rosen et al., 2010; Scholl et al., 2012).

## Conclusion

To summarize, we have assessed and characterized the homozygous ARTE10 mouse model for its feasibility for small animal FBB PET imaging and could demonstrate robust and progressive FBB signals, which correlated highly with actual amyloid pathology in the brain. The mouse model showed superior FBB retention in comparison to the APPswe/PS1ΔE9 line that nevertheless could be differentiated from wild-type animals. Our findings support the hypothesis that the total burden and number of congophilic dense-cored plaques are decisive factors for high FBB retention in the brains of amyloidosis models rather than technical difficulties, which could be managed by using appropriate reference regions for each mouse model.

## Data Availability Statement

The raw data supporting the conclusions of this article will be made available by the authors, without undue reservation.

## Ethics Statement

The animal study was reviewed and approved by the Landesamt für Natur, Umwelt und Verbraucherschutz Nordrhein-Westfalen (LANUV), North Rhine-Westphalia, Germany.

## Author Contributions

AW and K-JL planned and designed the study. MS, SS, PL, SBr, DH, NB, NJ, and SBe performed the experiments. JE provided the radiotracer with technical assistance. AW, MS, SS, and SBr performed the data analysis. AW wrote the manuscript. DW, NJS, and K-JL contributed to the manuscript. All authors contributed to the article and approved the submitted version.

## Conflict of Interest

The authors declare that the research was conducted in the absence of any commercial or financial relationships that could be construed as a potential conflict of interest.

## Publisher’s Note

All claims expressed in this article are solely those of the authors and do not necessarily represent those of their affiliated organizations, or those of the publisher, the editors and the reviewers. Any product that may be evaluated in this article, or claim that may be made by its manufacturer, is not guaranteed or endorsed by the publisher.
