# Supplementary material for: Comparison of the Amyloid Load in the Brains of Two Transgenic Alzheimer’s Disease Mouse Models Quantified by Florbetaben Positron Emission Tomography
Source: Front Neurosci. 2021 Oct 4;15:699926. doi: 10.3389/fnins.2021.699926 (PMC8520975; doi:10.3389/fnins.2021.699926)
Supplement: Supplementary file 1 [file Data_Sheet_1.pdf]

## Supplementary material

"Comparison of the Amyloid Load in the Brains of Two Transgenic Alzheimer´s Disease Mouse Models Quantified by Florbetaben Positron Emission Tomography"

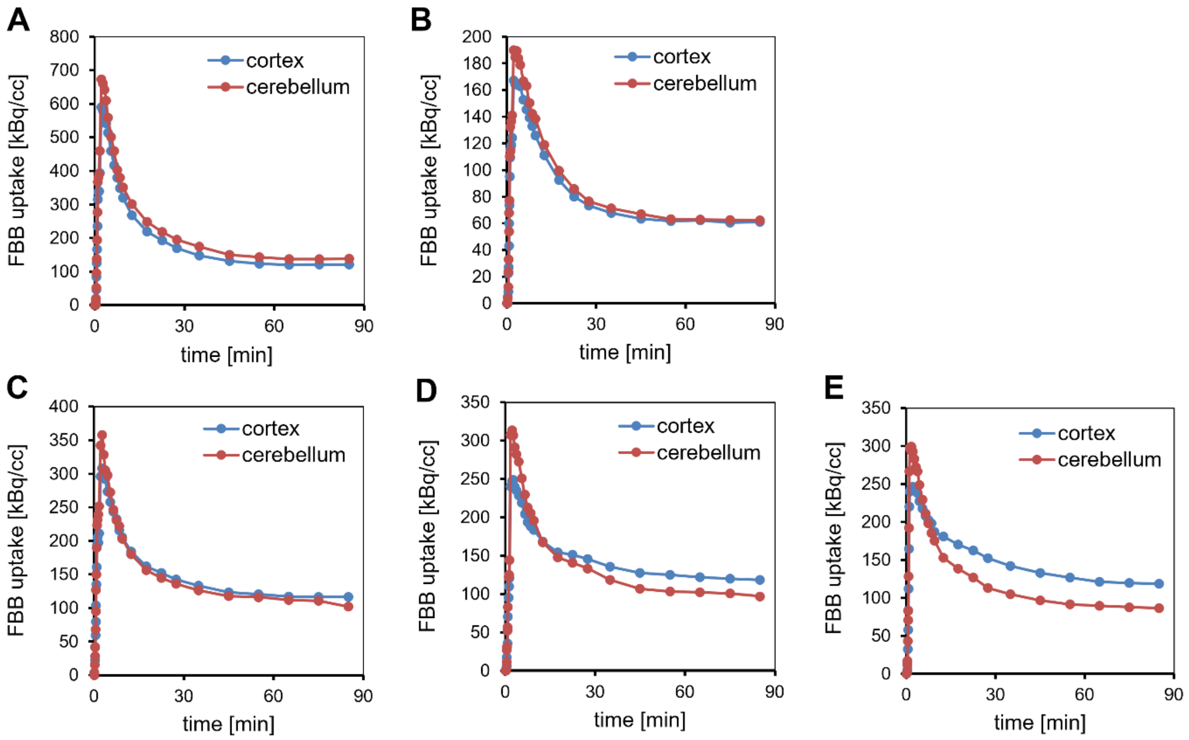


**Fig. S1. Time activity curves of florbetaben retention in the brains of ARTE10 and APPswe/PS1dΔE9 mice.**

Florbetaben (FBB) uptake and wash out in the brains of a representative wild-type mouse **(A)**, a 24-month-old APPswe/PS1dΔE9 mouse **(B)**, and homozygous ARTE10 mice at the age of 9 **(C)**, 15 **(D)** or 21 months **(E)** is depicted as time activity curves in cortical and cerebellar regions of interest. The same animals as in fig. 1 are shown.
